# Ring Finger Protein 213 in Moyamoya Disease With Pulmonary Arterial Hypertension: A Mini-Review

**DOI:** 10.3389/fneur.2022.843927

**Published:** 2022-03-24

**Authors:** Yuting Luo, Zhixin Cao, Shaoqing Wu, Xunsha Sun

**Affiliations:** ^1^Department of Neurology, National Key Clinical Department and Key Discipline of Neurology, The First Affiliated Hospital, Sun Yat-sen University, Guangzhou, China; ^2^Guangzhou Women and Children's Medical Center, Guangzhou Medical University, Guangzhou, China

**Keywords:** Moyamoya disease, pulmonary arterial hypertension, *RNF213*, idiopathic pulmonary arterial hypertension, peripheral pulmonary artery stenosis

## Abstract

Moyamoya disease (MMD), most often diagnosed in children and adolescents, is a chronic cerebrovascular disease characterized by progressive stenosis at the terminal portion of the internal carotid artery and an abnormal vascular network at the base of the brain. Recently, many investigators show a great interest in MMD with pulmonary arterial hypertension (PAH). *Ring finger protein 213 (RNF213)* is a major susceptibility gene for MMD and also has strong correlations with PAH. Therefore, this review encapsulates current cases of MMD with PAH and discusses MMD with PAH in the aspects of epidemiology, pathology, possible pathogenesis, clinical manifestations, diagnosis, and treatment.

## Introduction

Moyamoya disease (MMD) is a chronic and rare cerebrovascular disease characterized by an abnormal vascular network at the base of the brain and progressive stenosis or occlusion at the terminal part of the internal carotid artery and the initial part of the middle cerebral artery or the anterior cerebral artery. It is most common in East Asian populations, especially in Japanese, Korean, and Chinese. The MMD prevalence in Japan peaks at two ages: 5–10 and 25–49 years of age (1). The peak in the child group of MMD accounts for 16.2% of all incident cases, and the peak in the adult group accounts for 22.8% (2). The clinical presentations of MMD include transient ischemic attack (TIA), stroke, headache, epileptic seizures, and impaired mental function (3–6). Studies involving Asian populations indicate that adults with MMD have a much higher rate of hemorrhage stroke, whereas children with MMD have a higher rate of ischemic stroke or TIA (6). MMD has become one of the most important reasons for stroke in adolescents (7) and children (8), though it is an independent risk factor for recurrent stroke in children.

Recent studies found that MMD is not only related to intracranial vascular but is also related to some extracranial vascular, such as pulmonary arteries, renal arteries, and coronary arteries, among which the involvement of pulmonary arteries is common (9–11). Since 1990, Kapusta reported the first clinical case of MMD with pulmonary hypertension, more than 10 cases of MMD with pulmonary arterial hypertension (PAH) have been covered (12–20). PAH is a pathological condition in which pulmonary artery pressure is abnormally elevated with known and unknown etiology and can easily lead to right heart failure and even death as the disease progresses (21). Peripheral pulmonary artery stenosis (PPAS), with multiple stenosis and blockage of peripheral pulmonary arteries, can be seen in pediatric patients with congenital abnormalities or chromosomal syndromes (22–25). In addition, some cases of pulmonary hypertension for unknown reasons, called idiopathic pulmonary arterial hypertension (IPAH), are often associated with genetic factors (26).

The *Ring Finger Protein 213(RNF213)* on chromosome 17q25.3 has been identified as a susceptibility gene for MMD (27, 28). *RNF213* encodes a 591-kDa protein that possesses AAA+ ATPase domains and E3 ubiquitin ligase domains, which are closely related to various activities, such as angiogenesis, autophagy, autoimmunity, and lipid metabolism (29–32). *RNF213* p.R4810K has a strong relation with MMD in East Asian, which was detected in 95% of familial MMD cases (28) and 79% of sporadic cases (33). Other mutations are also associated with MMD in other areas in the world. On the other hand, a study has suggested a causal relationship between *RNF213* and PAH (34). *RNF213* p.R4810K was detected in 7.9% of patients with IPAH, resulting in a higher risk of lung transplantation and death and an earlier onset age (35). The incidence of PPAS in *RNF213* wild type, heterozygous p.R4810K, and homozygous p.R4810K MMD/quasi-MMD was 0% (0/101), 0.5% (1/200), and 40% (2/5), respectively (36). The positive detection effect of *RNF213* p.R4810k was no less than that of the recognized *bone morphogenic protein receptor type 2 (BMPR2)*, which is one of the causative genes in PAH (35). However, the relationship between *RNF213* and MMD with PAH is unknown. We summarized the current cases of MMD with PAH and discussed the epidemiology, pathology, possible pathogenesis, clinical manifestations, diagnosis, and treatment.

## Literature Search

We performed a search of the literature in the PubMed and Web of Science databases to identify articles related to MMD and PAH on November 20th, 2021. The titles and abstracts of those articles were reviewed by two reviewers to confirm their quality and eligibility for further examination. The inclusion criteria were as follows: (1) MMD and PAH, PPAS, or IPAH were simultaneously mentioned in the title or abstract, and (2) case reports. The exclusion criteria were as follows: (1) without a definite diagnosis of MMD and PAH, PPAS, or IPAH; (2) non-English article; and (3) Moyamoya syndrome. MMD was diagnosed when there was no specific underlying disease, including genetic, hereditary disorders, hematological disorders, connective-tissue diseases, infectious or chronic inflammatory conditions, metabolic diseases, and vascular injury in this study.

## Epidemiology and Clinical Manifestations

The prevalence of MMD with PAH is relatively high in East Asian countries (Table 1). Since the first clinical case of MMD with PAH was reported, we searched for patients with MMD accompanied by PAH and found 17 patients in 12 pieces of literature ranging from 1990 to 2021, excluding MMD syndrome and other patients with an unknown diagnosis. Among these cases, the minimum age at diagnosis of MMD was 1 year and the maximum age at diagnosis was 39 years. These cases have shown a younger age of MMD with PAH at diagnosis. Of the 14 patients with definite age of diagnosis, MMD often appears before the diagnosis of PAH (8/14), but sometimes appears simultaneously at the diagnosis of PAH (4/14). Additionally, the male-to-female ratio is 12:5, which is not consistent with the epidemiological phenomenon that MMD is predominant in women. The PPAS is most common in MMD with PAH, followed by IPAH. Seven patients died prematurely, with an average age of 15.71 years. Some patients died suddenly (14, 20), some patients died of postoperative complications like cerebral hernia or pneumonia (13), and some patients died for uncertain reasons.

**Table 1:** Case reports about patients with pulmonary hypertension and Moyamoya disease.

**Reference**		**Nationality**	**Sex**	**Age at diagnosis of MMD, year-old**	**Age of developing symptom of pulmonary hypertension, year-old**	**Etiology of PAH**	**mPAP/mmHg**	**Mutant gene**	**Family history of MMD (father/ mother)**	**Outcome**
Kramer et al. (19)	Patient 1	Germany	F	1	10	IPAH	44.00	Heterozygote RNF213 p.T4114I	N	12/alive
Ozaki et al. (36)	Patient 2	Japan	F	N	N	PPAS	N	Homozygote RNF213p.R4810K	N	19/alive
	Patient 3	Japan	M	N	N	PPAS	N	Homozygote RNF213 p.R4810K	N	56/alive
Takahashi et al. (14)	Patient 4	Japan	M	4	16	PPAS	63.00	Homozygote RNF213 p.R4859K	N	24/dead
Kizilkaya et al. (12)	Patient 5	Turkey	M	15	15	IPAH	80.00	N	N	16/alive
Chang et al. (20)	Patient 6	Korea	M	26	17	PAH	43.00	Homozygote RNF213p.R4810K	Heterozygote/ Heterozygote	26/alive
	Patient 7	Korea	F	3	19	PAH	62.00	Homozygote RNF213p.R4810K	Heterozygote/ Heterozygote	20/alive
	Patient 8	Korea	M	2	13	PPAS	46.00	N	N	13/dead
Ishiwata et al. (18)	Patient 9	Japan	M	39	31	PPAS	33.00	ELN	N	41/alive
Moceri et al. (37)	Patient 10	UK	M	13	21	PPAS	45.00	N	N	21/dead
Fukushima et al. (15)	Patient 11	Japan	M	9	13	PPAS	53.30	Homozygote RNF213p.R4810K	N	13/dead
	Patient 12	Japan	F	15	15	PPAS	64.67	Homozygote RNF213p.R4810K	N	15/alive
Schranz et al. (38)	Patient 13	Germany	M	10	N	PAH	101.00	N	N	19/dead
Tokunaga et al. (13)	Patient 14	Japan	M	14	14	PAH	32.67	N	N	14/dead
	Patient 15	Japan	F	2	4	PAH	N	N	N	6/dead
Ou et al. (39)	Patient 16	France	M	3	15	IPAH	54.00	N	N	15/alive
Kapusta et al. (16)	Patient 17	The Netherlands	M	5	5	IPAH	33.30	N	N	5/alive

*mPAP, mean pulmonary arterial pressure; F, female; M, male; MMD, Moyamoya disease; N, not available or uncertain; PPAS, peripheral pulmonary artery stenosis; IPAH, idiopathic pulmonary hypertension; PAH, pulmonary arterial hypertension; ELN, elastin; UK, The United Kingdom*.

Nine patients conducted genetic testing and up to 77.8% (7/9) of the patients carried homozygous *RNF213* p.R4810K mutation [*RNF213* p.R4859K and p.R4810K are identical, and p.R4810K is more widely used because it reflects the major transcript that lacks exon 4 (27, 31)]. One patient carried heterozygous *RNF213* p.T4114I mutation, and one patient carried *elastin (ELN)* mutation. The parents of two patients with homozygous p.R4810K mutation carried the heterozygous mutation, whereas the other family history remained unknown. *RNF213* p.R4810K is the most important variation in MMD, which is carried by about 80% of East Asians with MMD but is almost absent in European and American patients (27, 28, 40–43). This mutation is predominantly heterozygous in MMD, and homozygous mutations can be observed in 7–8% of patients (33, 44). In this study, the rate of homozygous p.R4810K mutation carrier in patients with MMD and PAH was significantly higher than that in patients with MMD alone. It is interesting that homozygous individuals with MMD and PAH were also found in Western populations. The mutations of *RNF213* p.R4810K can affect both the severity and presentation of the disease. The penetrance of MMD in heterozygous is 0.33–0.67%, whereas the incidence of MMD in homozygous is as high as 78% (44). The p.R4810K homozygous variant of *RNF213* predicted an early-onset, severe, and higher frequency of bilateral and posterior circulation form of MMD (33, 44, 45). Fukushima believed that the phenotype observed after *RNF213* mutation was gene-dosage-dependent manner (15). Heterozygous carriers of p.R4810K mostly presented MMD or PAH alone, whereas homozygous carriers were more likely to develop systemic vascular diseases including intracranial artery and peripheral artery like pulmonary artery involvement (15).

A recent whole-exome sequencing identified *RNF213* as the susceptibility gene for PAH (34). *RNF213* p.R4810K mutation has a higher positive detection effect than *BMPR2*, resulting in a higher risk of lung transplantation and death, an earlier onset age, less responsiveness to vasodilators, and a poor prognosis (35). PPAS was the most common cause of pulmonary hypertension in the patients included in this review (8/17), IPAH followed (4/17). PPAS may be underdiagnosed; thus, some patients with IPAH may have PPAS (23). The carrier rate of p.R4810K homozygous in patients with PPAS was much higher than heterozygous and wild type (36).

Thus, these results have suggested the importance of *RNF213* in MMD with PAH. In addition, several reports documented that *RNF213* gene mutations not only increase the risk of MMD but also are associated with intracranial atherosclerosis (46, 47) and systemic vascular diseases, such as PPAS (15, 20), renal artery stenosis (48), and coronary artery disease (49, 50). Therefore, MMD, PAH, and renal artery stenosis may be the specific manifestations of pathophysiological changes caused by *RNF213* mutation, which can be summarized as spectrums of *RNF213* vasculopathy (51, 52). The association between *RNF213* p.R4810K mutation and individual disease manifestation needs further study.

## Pathology

The pathological changes of MMD with PAH are partly similar to MMD or PAH. The typical pathological changes induced by MMD in the stenotic segment are thickening of the intima, medial thinness, and irregular undulation of the internal elastic laminae (53), with no infiltration of inflammatory cells and fats in the vascular wall (54). However, the thickened intima was composed predominantly of smooth muscle cells with an admixture of some macrophages and T-cell scattering in the superficial layer of the intimal thickening (55). Marked perivascular inflammation was present in a high number of PAH lungs and correlated with intima plus media remodeling (56). There are two cases of IPAH (or hereditary) carrying heterozygous *RNF213* p.R4810K (35). Pathological examination of the first patient indicated plexiform and concentric neointimal lesions in the pulmonary arteries. In the second case, pathologic examination demonstrated marked venous wall thickness and venous occlusion accompanied by dilation of capillary vessels and lymphedema of the interlobular septa, and arterial wall thickening. PPAS has been widely reported in clinical cases of MMD with PAH (14–16, 20). Takahashi et al. (14) recently reported a case of MMD with PPAS with homozygous *RNF213* p.R4810K. Autopsy results showed the proximal pulmonary artery was dilated (the diameter of the pulmonary valve was 65 mm), and multiple stenoses in the branch pulmonary arteries with post-stenotic dilation. In this case, the membrane became thicker and the inner elastic lamina showed continuous and irregular waving, compared with patients with MMD. There are significant differences among the internal elastic layer between the patients with MMD and PAH and patients with PPAS, even though they share the same pathologic changes in intima and media. The irregular waving of the internal elastic (57) is the pathological feature that is not present in PPAS. As the family of the patient refused craniotomy, it is unknown whether the patient's cerebrovascular pathology showed similar pathological changes with the pulmonary artery. Thus, existing pathological results suggest that the mutation of the *RNF213* p.R4810K has an important influence on the pathology of MMD with PAH, and further exploration with more samples is required.

## Pathogenesis Underlying MMD With PAH

### *RNF213* and Angiogenesis

The abnormal angiogenesis has a strong association with the development of MMD (58, 59) and PAH (60, 61). Recent studies have suggested the importance of *RNF213* in the pathogenesis of MMD with PAH (14, 35). Mysterin, encoded by *RNF213* containing enzymatically active AAA+ ATPase and E3 ubiquitin ligase domains, has a complex structure and function, which can participate in several physiological activities in cells, especially plays an important role in angiogenesis. Ito et al. (62) found that angiogenesis in *RNF213* knockout mice was enhanced after chronic hind-limb ischemia, suggesting that abnormal *RNF213* may be involved in the development of the vascular network in chronic ischemia. The p.R4810K mutation is located in the E3 core at the C-terminal of the *RNF213* and affects the function of vascular endothelial cells (63). Meanwhile, Kobayashi et al. (57) found that *RNF213* p.R4810K had antiangiogenic activity through decreasing ATPase activity to stabilize oligomers. *In vitro* experiments, endothelial cells derived from pluripotent stem cells with *RNF213* p.R4810K induced significantly lower angiogenesis activity than wild-type cells (64). Further studies showed that *RNF213* p.R4810K induced downregulation of *securin* (64). However, it remains unable to have a comprehensive and systematic explanation for the abnormal angiogenesis of MMD with PAH. We need more investigations to provide an overall insight into their mechanism.

### *RNF213* and Caveolin-1

Caveolin-1 (Cav-1), the signature protein of endothelial cell caveolae, is closely related to the function of endothelial cells and smooth muscle cells (65, 66). Caveolins participate in many important cellular processes, including vesicular transport, cholesterol homeostasis, signal transduction, and tumor suppression (66). In patients with MMD and homozygous *RNF213*, p.R4810K mutation, Cav-1 positive expression of *RNF213* was found in the thickened intima (14). Recently, researchers found that the expression of Cav-1 in plasma of patients with MMD was decreased, especially in patients carrying *RNF213* p.R4810K (30). The decrease of Cav-1 was positively correlated with the narrowing of the distal external diameter of the internal carotid artery in adult patients with MMD (59). *In vitro* study, the downregulation of Cav-1 expression could induce apoptosis in endothelial cells and lead to lumen formation disorder, which may play a role in the reduction and remodeling of intracranial arterial external diameter (59). In patients with IPAH, Cav-1 protein was reduced in human pulmonary artery endothelial cells (67) but increased in human pulmonary smooth muscle cells (68). Serum Cav-1 level was also significantly decreased in IPAH (69). Cav-1 knockout mice have some prominent features including adverse lung phenotype with thickened alveolar septa, smaller alveolar spaces, and an interstitial hypercellularity, and it is further characterized by substantial PAH accompanied by a hypertrophied right ventricle (70–73). Moreover, Kobayashi, H et al. found that increased ventricular pressure, end-diastolic ventricular diameter ratio, pulmonary vascular muscularization, ablation of pulmonary vascular endothelial cells from the basal membrane, and decreased Cav-1 in mice with *RNF213* p.R4810K gene mutation under hypoxia (74). Meanwhile, a study demonstrated that the artery stiffness increased in Cav-1deficient mice, especially the circumferential stiffness of the pulmonary arteries (75). The mutation of the *RNF213* p.R4810K may affect endothelial cell function by interference with the Cav-1. In human pulmonary smooth muscle cells of PAH, Cav-1 may influence intracellular calcium concentration (68, 76) and cytoplasmic vesicle transport (77). In MMD with PAH, *RNF213* p.R4810K may affect vascular smooth muscle cells *via* Cav-1. The relationship between *RNF213* and Cav-1 will provide a new direction for us to explore the pathogenesis of MMD with PAH. In a word, the mutation of the *RNF213* p.R4810K is an important factor leading to pathological abnormalities of PAH in MMD, but the specific mechanism of the effect on pulmonary vascular and cerebrovascular needs to be further studied.

### Other Factors

In some cases of MMD with PAH, pulmonary angiography suggests peripheral pulmonary stenosis with beading and bending (15, 20, 39). Arterial beaded lesions are an important feature of fibromuscular dysplasia (78). Fibromuscular dysplasia is a rare non-atherosclerotic and non-inflammatory arterial disease that mainly involves small and medium arteries. There are two patients with homozygous *RNF213* p.R4810K mutations accompanying MMD with bead-like pulmonary angiography (15, 20). Fibromuscular dysplasia (FMD) may be the basis of intracranial and extracranial vascular lesions in MMD. Recently, a study reported that *RNF213* rare coding variants suggested a gene-based association with multifocal FMD (79). However, p.R4810k was not present in the exome array that generated genotypes in FMD cases (79). Therefore, the relationship between *RNF213* other gene mutations and FMD needs further study. In addition, a study reported a clinical case of *ELN* mutation in MMD with PAH. Elastic fibers play an important role in maintaining the elasticity of tissues, such as arteries, lungs, and skin. Elastic fiber abnormality can cause a variety of cardiovascular and skin diseases, such as descending aortic stenosis, descending aortic stenosis, and skin laxity (80–82). This suggests that abnormal vascular wall components may lead to systemic vascular diseases, such as MMD, PAH, renal artery stenosis, and other symptoms.

## Diagnosis and Treatment

When young patients, with a clear history of MMD, show dyspnea and/or syncope, we should be vigilant for PAH (14, 15, 19, 20, 36). In the same way, when the patient with PAH showed unprovoked and spontaneously recurring epileptic seizures, movement disorder, and retardation of psychomotor development, we should be suspicious about MMD (12, 19). The patients with MMD and PAH did not show any specific signs in physical examination (20). From a clinical standpoint, cerebrovascular or pulmonary vascular investigations may be warranted in patients with PAH or MMD, respectively. As MMD and PAH have their complete diagnostic criteria, respectively, it's not difficult to have definite diagnoses for both diseases. In patients with MMD with *RNF213* p.R4810k, they can accompany PAH (35). Thus, regular echocardiographic screening for early signs of PAH in patients with MMD should be part of regular clinical workup (19). *RNF213* gene mutations, especially p.R4810k are associated with various intracranial and extracranial vascular lesions, such as intracranial artery stenosis/occlusion disease (83, 84), intracranial atherosclerosis (85), PPAS (15, 20), renal artery stenosis (48), coronary artery disease (49, 50), superior mesenteric arteries stenosis (86), and so on (87). Therefore, if patients with MMD had a homozygous (or compound heterozygous) *RNF213* mutation, systemic screening may be useful. In this way, early detection and treatment of MMD with PAH might help to improve the long-term outcome and quality of life.

As the treatment of MMD with PAH, there is still in the exploratory stage. Some scholars suggest patients with MMD and PAH should be treated as early as possible with dual/triple therapy (19). However, the therapeutic effect of drugs on MMD with PAH is not obvious (12, 20), and there are not enough case studies. At the same time, there is a controversy about the application of angioplasty in MMD with PAH (20, 37). Vasculopathy in MMD involves intimal hyperplasia of smooth muscle cells and possibly results in immediate elastic recoil or progressive restenosis, which has been noted during angioplasty of cerebral arteries in patients with MMD (88). As MMD with PAH shares the same pathologic changes in intima and media with MMD, the same thing may happen in the patients of MMD with PAH (15). Hence, we should be cautious when treating these patients with MMD and PAH with percutaneous angioplasty. A palliative Potts shunt may be a good choice for end-stage patients with MMD and PAH (38). Thus, more studies on MMD with PAH are needed to find an effective treatment for the disease.

## Conclusion

In conclusion, we have reviewed that *RNF213* plays an important role in MMD with PAH at epidemiology, pathology, possible pathogenesis, clinical manifestations, clinical manifestations, diagnosis, and treatment. Most patients with MMD complicated with PAH carried *RNF213* p.R4810K and were accompanied by mutation-associated pathophysiological changes. Thus, the detection of *RNF213* mutation, especially p.R4810K, could be used as a part of the etiology study for MMD with PAH. At the same time, *RNF213* p.R4810K may be a predictor for systemic vascular examination. Although it is unable to have a comprehensive and systematic explanation for the occurrence and development of MMD with PAH, we can regard *RNF213* as a target to better understand the possible pathogenesis of this disease. In particular, significant research will have to be undertaken to uncover the relationship between *RNF213* and vascular disease, which is not limited to MMD.

## Author Contributions

XS and SW designed this review. YL drafted the manuscript. YL and ZC searched and reviewed the database and all included articles and reviewed the articles based on inclusion and exclusion criteria. XS, SW, and YL provided comments and revised the manuscript. All authors have approved the final version of the manuscript.

## Funding

This study was funded by the National Natural Science Foundation of China (82071292, 81601004, and 81102248), Kelin New Talent Program (R08016), the Natural Science Foundation of Guangdong Province (2016A030310139), the Fundamental Research Funds for the Central Universities (20ykpy66), the Guangdong Provincial Key Laboratory of Diagnosis and Treatment of Major Neurological Diseases (2020B1212060017), the Guangdong Provincial Clinical Research Center for Neurological Diseases (2020B1111170002), the Southern China International Cooperation Base for Early Intervention and Functional Rehabilitation of Neurological Diseases (2015B050501003 and 2020A0505020004), the Guangdong Provincial Engineering Center for Major Neurological Disease Treatment, and the Guangdong Provincial Translational Medicine Innovation Platform for Diagnosis and Treatment of Major Neurological Disease.

## Conflict of Interest

The authors declare that the review was conducted in the absence of any commercial or financial relationships that could be construed as a potential conflict of interest.

## Publisher's Note

All claims expressed in this article are solely those of the authors and do not necessarily represent those of their affiliated organizations, or those of the publisher, the editors and the reviewers. Any product that may be evaluated in this article, or claim that may be made by its manufacturer, is not guaranteed or endorsed by the publisher.
